# Supplementary material for: Non-canonical peptidoglycan cross-linking is essential for Mycobacterium tuberculosis acid resistance
Source: bioRxiv. 2026 Mar 16:2026.03.16.712108. Preprint. [Version 1] doi: 10.64898/2026.03.16.712108 (PMC13015542; doi:10.64898/2026.03.16.712108)
Supplement: Supplement 2 [file NIHPP2026.03.16.712108v1-supplement-2.pdf]

bioRxiv preprint doi: https://doi.org/10.64898/2026.03.16.712108; this version posted March 16, 2026. The copyright holder for this preprint (which was not certified by peer review) is the author/funder, who has granted bioRxiv a license to display the preprint in perpetuity. It is made available under aCC-BY 4.0 International license.

Supplementary Figures

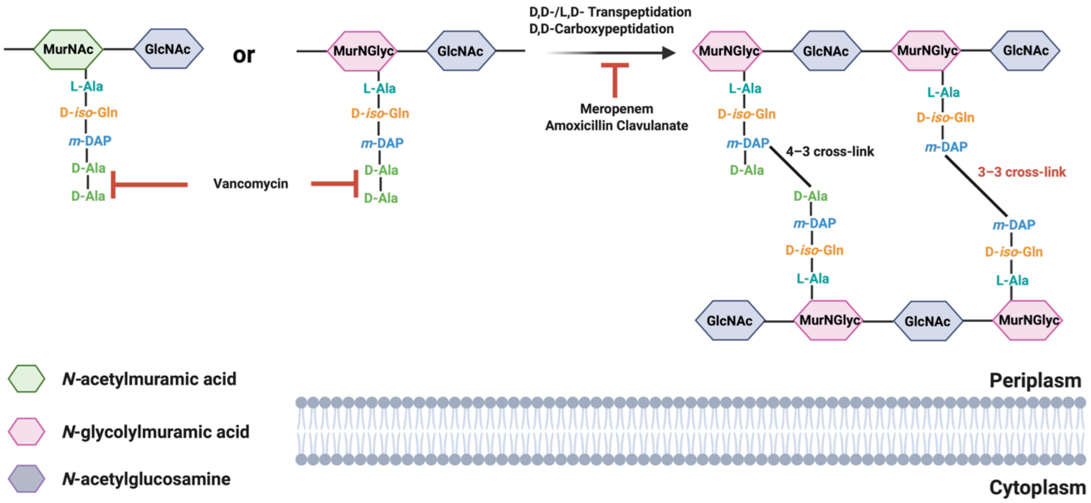

Supplementary Fig. 1: Schematic representation of *M. tuberculosis* peptidoglycan structure and the inhibition sites of PG-targeting antibiotics. Peptidoglycan (PG) subunits composed of alternating N-acetylglucosamine (GlcNAc) and either N-acetylmuramic acid (MurNAc) or N-glycolylmuramic acid (MurNGlyc) carry a conserved stem peptide (L-Ala–D-iso-Gln–meso-diaminopimelic acid [m-DAP]–D-Ala–D-Ala). In the periplasm, stem peptides are cross-linked either via D,D-transpeptidation to form 4–3 cross-links or via L,D-transpeptidation to form 3–3 cross-links. DDTs cleave the terminal D-alanine residue from the donor stem to form a cross-link between the fourth amino acid (D-Ala) of the donor stem and the third amino acid (meso-diaminopimelate, meso-DAP) of the acceptor stem. LDTs cleave the terminal (fourth) D-alanine from the donor stem and catalyze 3–3 cross-linking by joining the meso-DAP residues at the third position of both the donor and acceptor peptide stems. D,D-carboxypeptidation removes the terminal D-Ala to generate tetrapeptide substrates for L,D-transpeptidases. β-lactam antibiotics such as meropenem and amoxicillin clavulanate inhibit transpeptidation and D,D-carboxypeptidation, whereas vancomycin binds the D-Ala–D-Ala motif of pentapeptide stems, blocking downstream cross-link formation. Colored symbols denote individual sugar residues and amino acids as indicated.

bioRxiv preprint doi: https://doi.org/10.64898/2026.03.16.712108; this version posted March 16, 2026. The copyright holder for this preprint (which was not certified by peer review) is the author/funder, who has granted bioRxiv a license to display the preprint in perpetuity. It is made available under aCC-BY 4.0 International license.

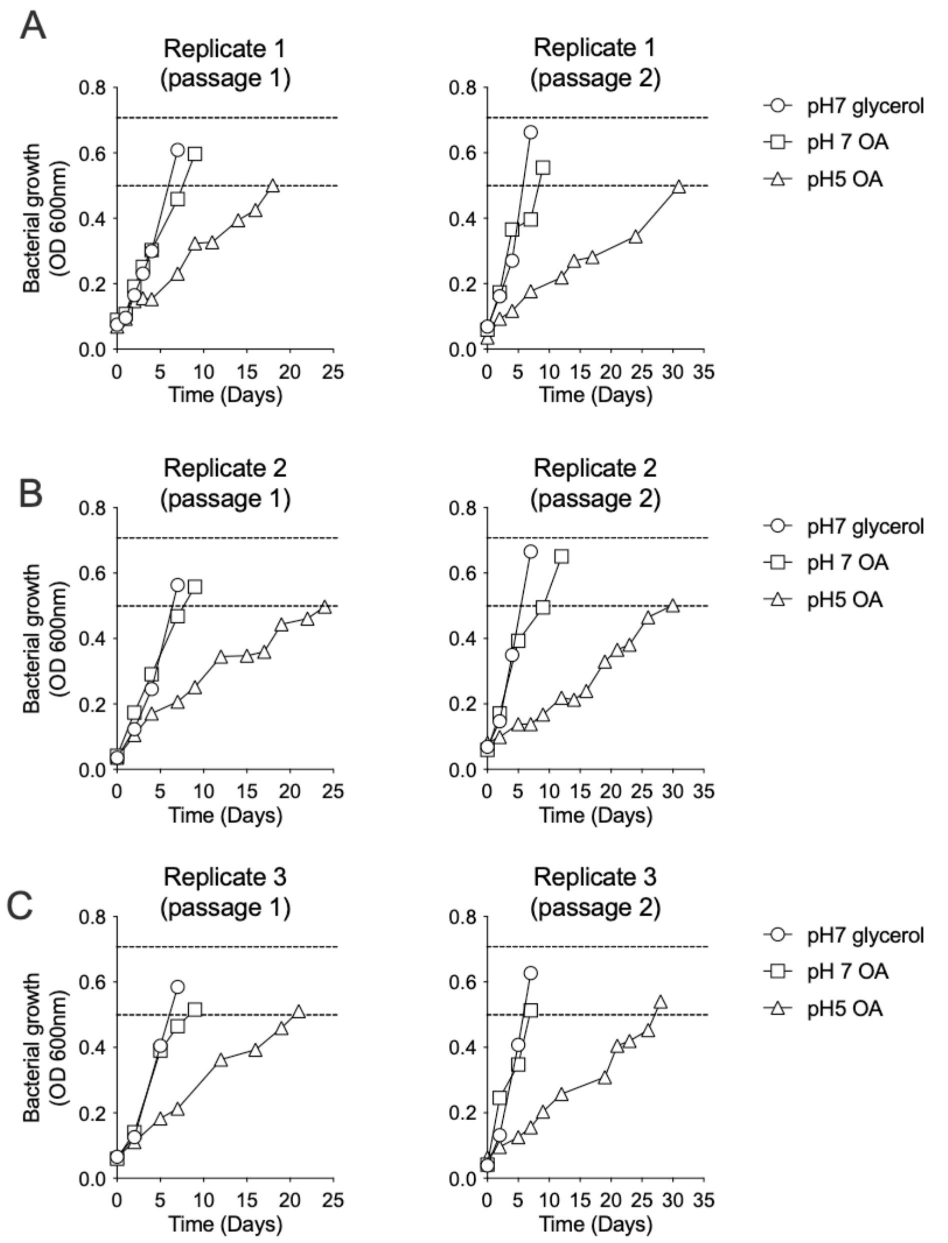

Supplementary Fig. 2: Growth patterns of the *M. tuberculosis* H37Rv transposon library under different carbon sources and pH conditions. (A) Growth curves for replicate 1 after first (left) and second passage (right). **(B)** Growth curves for replicate 2 after first (left) and second passage (right). **(C)** Growth curves for replicate 3 after first (left) and second passage (right). For all replicates and passages, bacteria were harvested in mid-log phase (OD= 0.5-0.7).

bioRxiv preprint doi: https://doi.org/10.64898/2026.03.16.712108; this version posted March 16, 2026. The copyright holder for this preprint (which was not certified by peer review) is the author/funder, who has granted bioRxiv a license to display the preprint in perpetuity. It is made available under aCC-BY 4.0 International license.

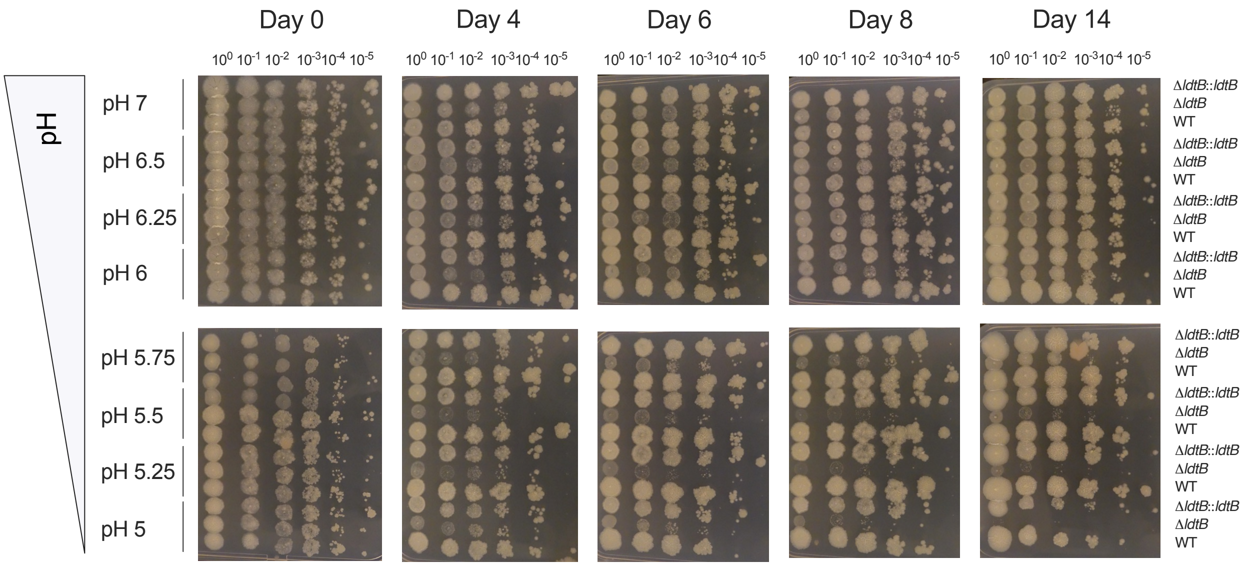

Supplementary Fig. 3: 7H10 plate images of spot assays. WT, Δ*ldtB*, and Δ*ldtB::ldtB* strains were grown at various pH and serial dilutions of each culture were spotted overtime onto plates on days 0, 4, 6, 8, and 14. Data are representative of three independent experiments.

bioRxiv preprint doi: https://doi.org/10.64898/2026.03.16.712108; this version posted March 16, 2026. The copyright holder for this preprint (which was not certified by peer review) is the author/funder, who has granted bioRxiv a license to display the preprint in perpetuity. It is made available under aCC-BY 4.0 International license.

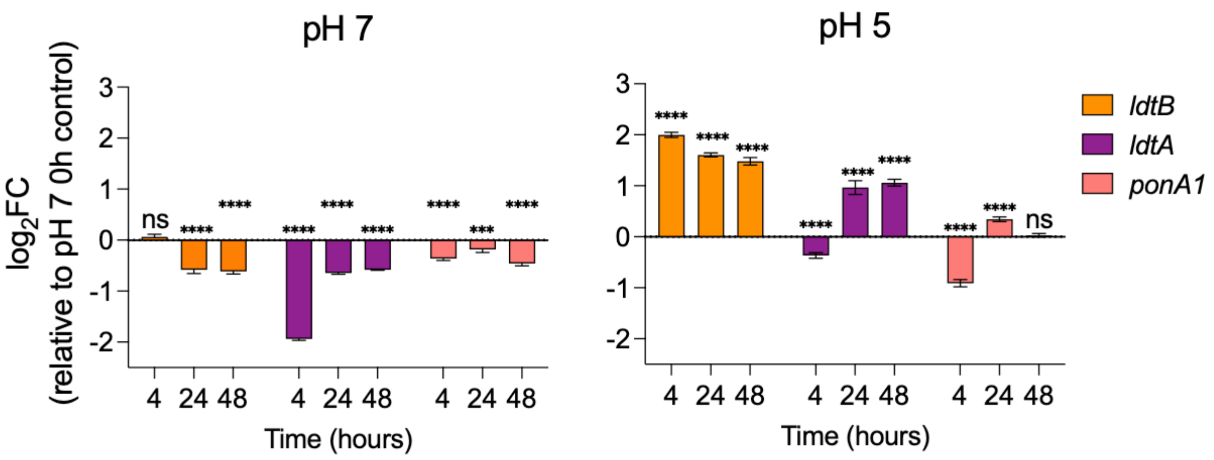

Supplementary Fig. 4: Expression of *ldtA*, *ldtB*, and *ponA1* in 7H9 medium supplemented with oleic acid (OA) at pH 7 or pH 5. Gene expression levels are presented as $\log_2$ fold change relative to the 0 h time point at pH 7 and are shown as mean ± SD from three independent biological replicates (n = 3). Statistical significance was determined using two-way ANOVA with Dunnett's multiple comparisons versus 0 h within each pH; ns, not significant; ***, adjusted $p < 0.001$; ****, adjusted $p < 0.0001$.

bioRxiv preprint doi: https://doi.org/10.64898/2026.03.16.712108; this version posted March 16, 2026. The copyright holder for this preprint (which was not certified by peer review) is the author/funder, who has granted bioRxiv a license to display the preprint in perpetuity. It is made available under aCC-BY 4.0 International license.

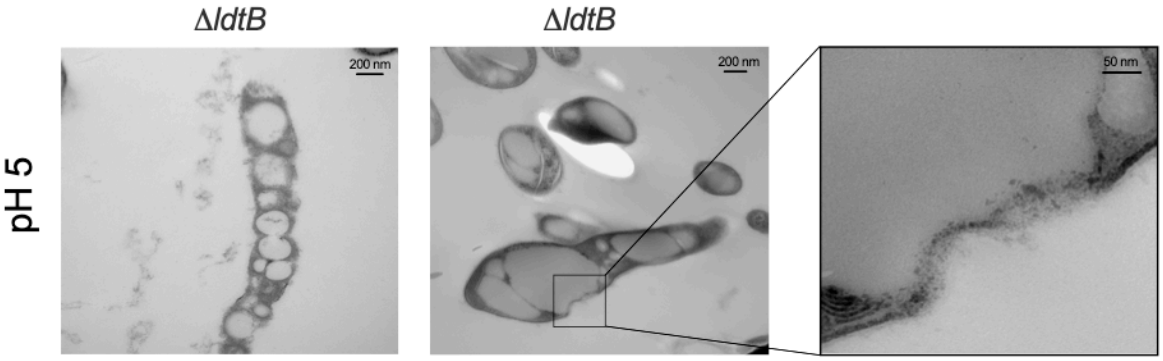

Supplementary Fig. 5: Transmission electron micrographs of Δ*ldtB* cells grown for 8 days at pH 5. Acid-specific cell-envelope defects are observed in the Δ*ldtB* mutant. Scale bars for 200nm or 50 nm are shown at the top right of each micrograph; accelerating voltage used was 100kv and magnification was 50,000x (left panel), 40,000x (middle panel) and 250, 000x (right panel).

bioRxiv preprint doi: https://doi.org/10.64898/2026.03.16.712108; this version posted March 16, 2026. The copyright holder for this preprint (which was not certified by peer review) is the author/funder, who has granted bioRxiv a license to display the preprint in perpetuity. It is made available under aCC-BY 4.0 International license.

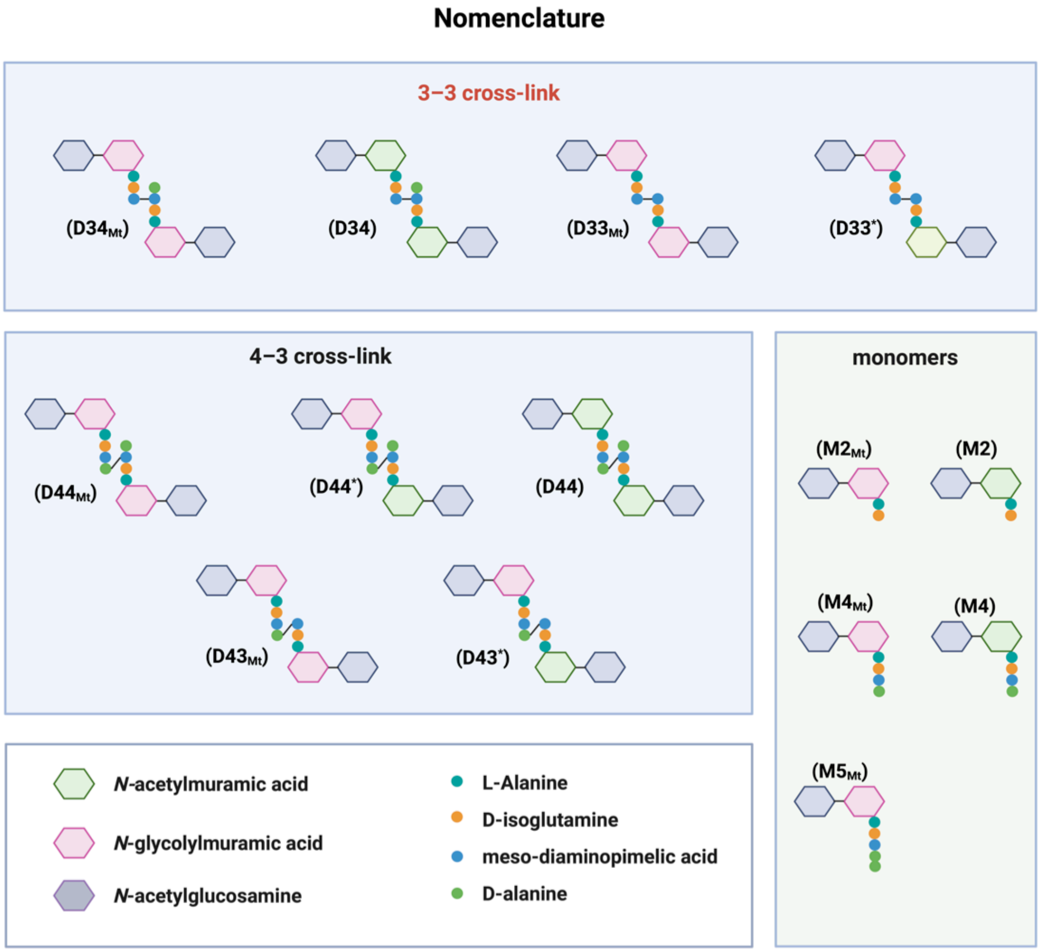

Supplementary Fig. 6: Peptidoglycan muropeptide nomenclature. PG fragments from *M. tuberculosis* were annotated using a letter–number system, where M, D, and T denote monomers, dimers, and trimers, respectively, and numbers indicate peptide stem length (donor–acceptor for cross-linked species). The suffix "Mt" denotes fully N-glycolylated species, an asterisk (*) indicates dimers containing mixed N-glycolylated and N-acetylated muramic acid, and unlabeled species are fully N-acetylated. L,D- and D,D-transpeptidase–derived 3–3 and 4–3 cross-links were distinguished by stem length and linkage chemistry, and anhydro-muramic acid was denoted by "N".

bioRxiv preprint doi: https://doi.org/10.64898/2026.03.16.712108; this version posted March 16, 2026. The copyright holder for this preprint (which was not certified by peer review) is the author/funder, who has granted bioRxiv a license to display the preprint in perpetuity. It is made available under aCC-BY 4.0 International license.

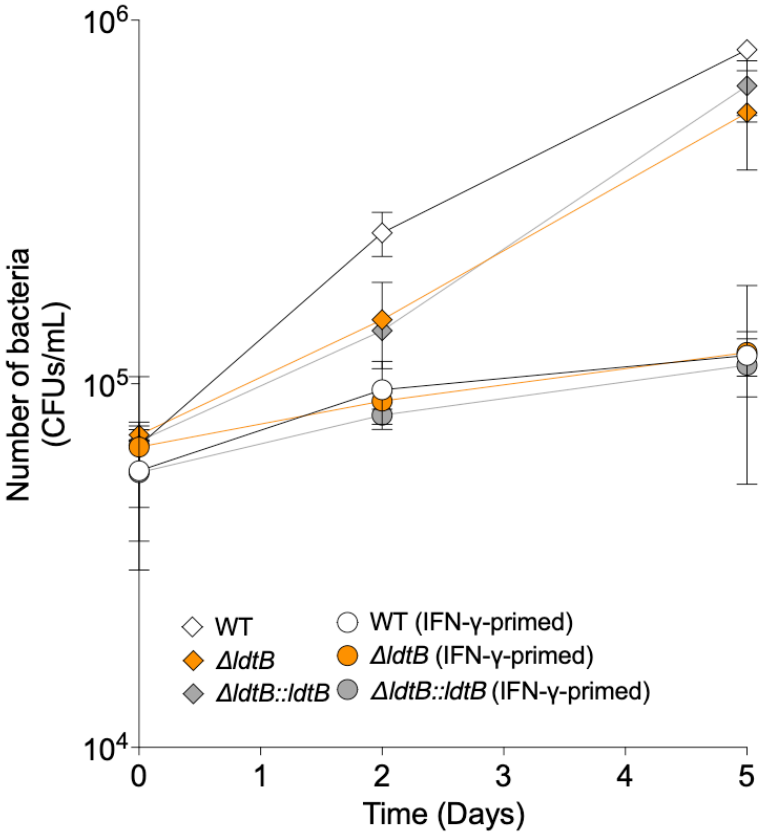

Supplementary Fig. 7: Survival of WT, Δ*ldtB*, and Δ*ldtB::ldtB* strains in BMDMs. Intracellular bacterial burden was quantified by CFU enumeration at the indicated time points over a 5-day infection period, either in the absence or presence of IFN-γ activation. Statistical significance was determined using one-way ANOVA with Tukey's multiple comparisons test. No significant differences in intracellular survival were observed among the three strains under either condition. CFU data represent mean ± SD from three independent wells per condition and are representative of three independent experiments.

bioRxiv preprint doi: https://doi.org/10.64898/2026.03.16.712108; this version posted March 16, 2026. The copyright holder for this preprint (which was not certified by peer review) is the author/funder, who has granted bioRxiv a license to display the preprint in perpetuity. It is made available under aCC-BY 4.0 International license.

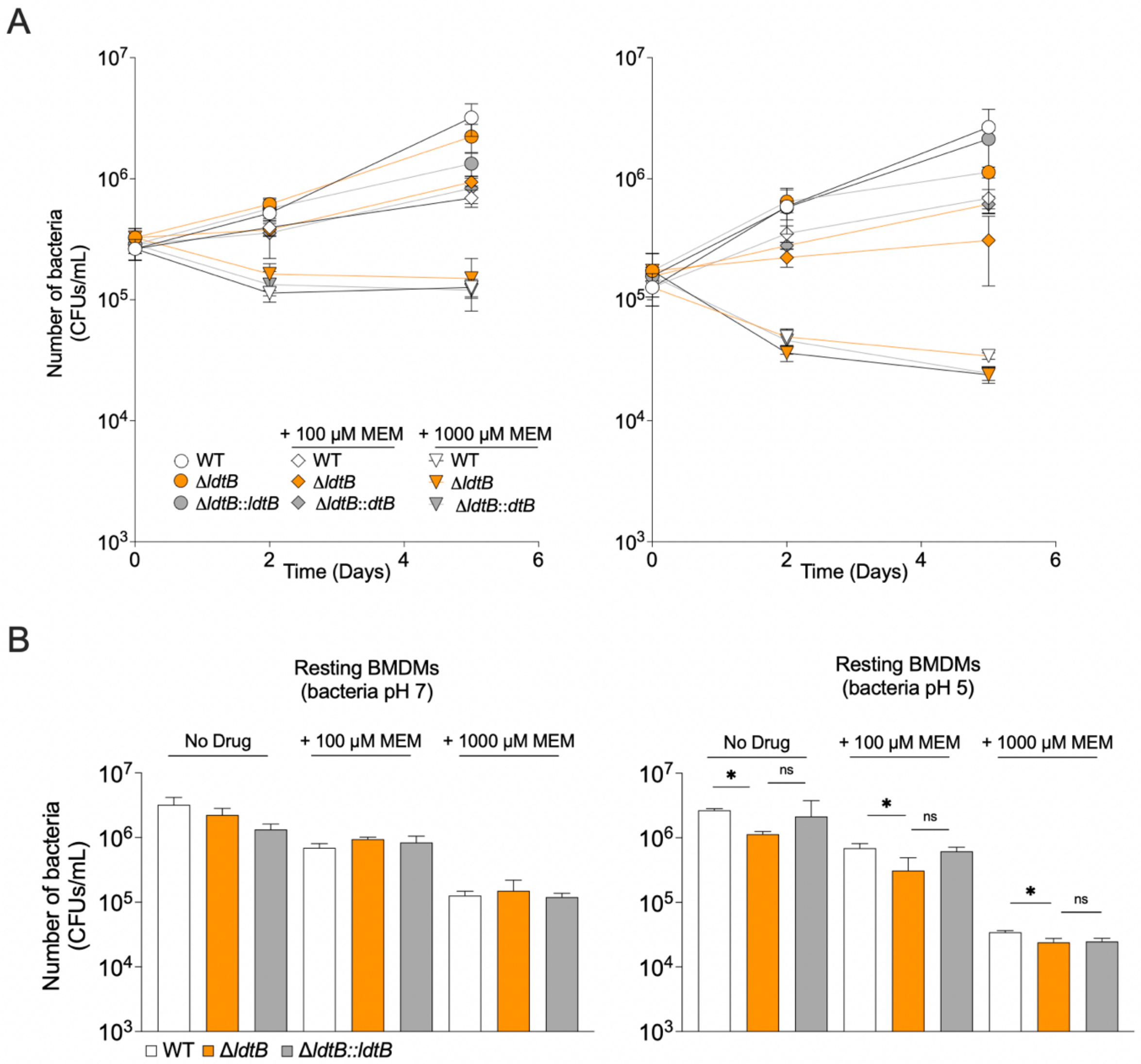

Supplementary Fig. 8: Survival of preconditioned WT, Δ*ldtB*, and Δ*ldtB::ldtB* strains in resting BMDMs treated with increasing concentrations of meropenem. Intracellular bacterial burden was quantified by CFU enumeration on days 0, 2, and 5. CFU values on day 5 were statistically compared between strains. Statistical significance was determined using one-way ANOVA with Tukey's multiple comparisons test; ns, not significant; **, adjusted $p < 0.01$; ***, adjusted $p < 0.001$. CFU data represent mean ± SD from three independent wells per condition and are representative of three independent experiments.

bioRxiv preprint doi: https://doi.org/10.64898/2026.03.16.712108; this version posted March 16, 2026. The copyright holder for this preprint (which was not certified by peer review) is the author/funder, who has granted bioRxiv a license to display the preprint in perpetuity. It is made available under aCC-BY 4.0 International license.

Supplementary Table 1:

Target	Forward Primer	Reverse Primer	Length	Tm	Probe	Labeling
ldtA	GCAGGAACTGACCGAAGGAT	TCTGCTGACCGTGAAGGTAT	20	65.48	TCGAGACCGGTGACGCACTGAT	FAM-BHQ1
ldtB	AACGCGCAGTGGTTCTACGA	TGCCACCCACGGTATTGAC	19	67	TGTCAAGCGCGGCGACATCGT	FAM-BHQ1
ponA1	CGGTCGACGGCATCAAGA	TTGAGCATCAGCCGGTAGTAG	21	66.28	TCACCAACGTCGAGGGCGAG	FAM-BHQ1
